# Supplementary material for: Robust and Scalable Angiogenesis Assay of Perfused 3D Human iPSC-Derived Endothelium for Anti-Angiogenic Drug Screening
Source: Int J Mol Sci. 2020 Jul 7;21(13):4804. doi: 10.3390/ijms21134804 (PMC7370283; doi:10.3390/ijms21134804)
Supplement: Supplementary file 1 [file ijms-21-04804-s001.pdf]

Supplementary Material

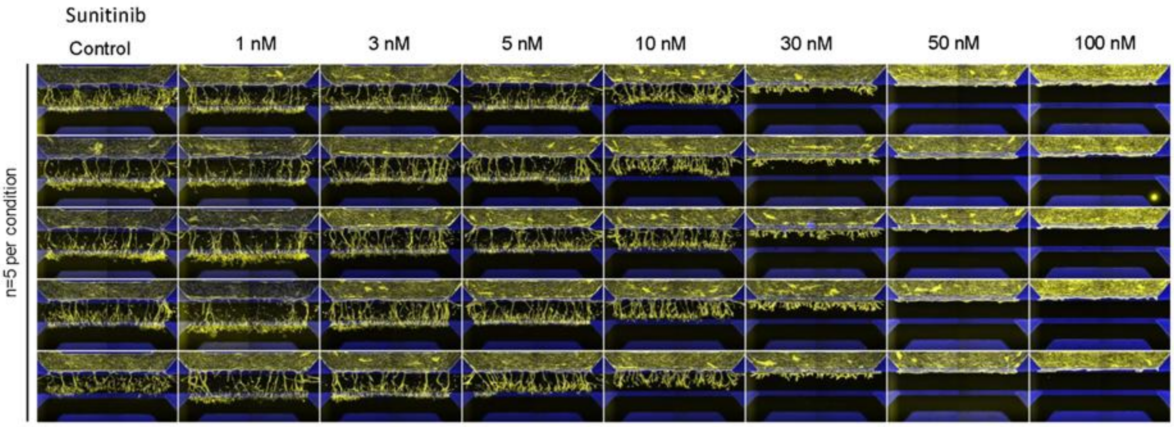

Figure S1. Concentration optimization study for Sunitinib in a single assay. Inhibition of angiogenic sprouting of iPSC-ECs using various concentrations of Sunitinib. 40 microfluidic units are distributed over 8 conditions with each five replicates and shows sprouting is reproducible within a single plate.
